# Nutrient Limitation in Three Lowland Tropical Forests in Southern China Receiving High Nitrogen Deposition: Insights from Fine Root Responses to Nutrient Additions

**DOI:** 10.1371/journal.pone.0082661

**Published:** 2013-12-20

**Authors:** Feifei Zhu, Muneoki Yoh, Frank S. Gilliam, Xiankai Lu, Jiangming Mo

**Affiliations:** 1 Key Laboratory of Vegetation Restoration and Management of Degraded Ecosystems, South China Botanical Garden, Chinese Academy of Sciences, Guangzhou, China; 2 United Graduate School of Agriculture Science, Tokyo University of Agriculture and Technology, Tokyo, Japan; 3 Institute of Agriculture, Tokyo University of Agriculture and Technology, Tokyo, Japan; 4 Department of Biological Sciences, Marshall University, Huntington, West Virginia, United States of America; 5 University of Chinese Academy of Sciences, Beijing, China; Bangor University, United Kingdom

## Abstract

Elevated nitrogen (N) deposition to tropical forests may accelerate ecosystem phosphorus (P) limitation. This study examined responses of fine root biomass, nutrient concentrations, and acid phosphatase activity (APA) of bulk soil to five years of N and P additions in one old-growth and two younger lowland tropical forests in southern China. The old-growth forest had higher N capital than the two younger forests from long-term N accumulation. From February 2007 to July 2012, four experimental treatments were established at the following levels: Control, N-addition (150 kg N ha^–1^ yr^–1^), P-addition (150 kg P ha^–1^ yr^–1^) and N+P-addition (150 kg N ha^–1^ yr^–1^ plus 150 kg P ha^–1^ yr^–1^). We hypothesized that fine root growth in the N-rich old-growth forest would be limited by P availability, and in the two younger forests would primarily respond to N additions due to large plant N demand. Results showed that five years of N addition significantly decreased live fine root biomass only in the old-growth forest (by 31%), but significantly elevated dead fine root biomass in all the three forests (by 64% to 101%), causing decreased live fine root proportion in the old-growth and the pine forests. P addition significantly increased live fine root biomass in all three forests (by 20% to 76%). The combined N and P treatment significantly increased live fine root biomass in the two younger forests but not in the old-growth forest. These results suggest that fine root growth in all three study forests appeared to be P-limited. This was further confirmed by current status of fine root N:P ratios, APA in bulk soil, and their responses to N and P treatments. Moreover, N addition significantly increased APA only in the old-growth forest, consistent with the conclusion that the old-growth forest was more P-limited than the younger forests.

## Introduction

Many tropical/subtropical regions, eastern Asia in particular, are receiving high levels of atmospheric nitrogen (N) deposition, with such increases projected to continue in the next decades [1], [2]. This will add more new N to some lowland tropical forests and may alter soil nutrient availability and carbon (C) balance of these ecosystems. In general, humid tropical forests store approximately 10% of global soil C. Thus, it is crucial to know the current nutrient status of these ecologically important forests and how their structure and function may be altered by increased N deposition [3].

Plant growth in lowland tropical forest is generally considered to be limited by phosphorus (P), rather than N [4], [5], [6]. Further N inputs from atmospheric deposition are likely to alter soil nutrient conditions negatively, including increases in soil acidification, losses of base cations, and Al mobilization [7], [8], [9], [10], which consequently may lower soil P availability [7], [11]. These predictions have been observed in some temperate forests where locally-elevated N deposition contributes to a transition in nutrient limitation to forest growth, i.e., driving forests from N limitation to N saturation and P limitation [12], [13], [14]. However, such effects of N deposition on ecosystem nutrient limitation have rarely been investigated in tropical forests [15].

Responses of fine root growth to N or P limitation are inherently different, due to the contrasting mobility of nitrate (NO_3_) and phosphate (PO_4_) in soils [16]. NO_3_ is very mobile in the soil and approaches the root via mass flow. Accordingly, new root production may not be required for plants to absorb additional N. Nitrogen limitation ceases when N availability increases beyond N demand. Subsequently, less fine root biomass is needed, according to the allocation theory proposed by Bloom et al. 1985 [17]. Decreases in fine root biomass are frequently observed as N availability increases further [18], [19]. In contrast, PO_4_ is fairly immobile due to adsorption by organic matter and Al- and Fe-oxides in highly weathered soils. Roots encounter P only by growing through soil rather than having P delivered via diffusion or water flow. As a result, fine roots growing in P-limited soils will theoretically respond to additional P by more growth [20]. Thus, less fine root biomass following N additions is generally expected, whereas more fine root biomass following P additions can imply P limitation.

As indirect proxies for nutrient limitation, N and P concentrations in fine roots have received less attention than those of leaves [21]. It is suggested that leaves have a stronger homeostatic control over N:P ratios than roots [22]. Thus, nutrient concentrations and N:P ratios of fine roots should respond more sensitively to changes in soil nutrient availability. In addition to fine root responses, acid phosphatase, the dominant form of extracellular phosphatase in acid organic-rich soils, has also been suggested to be a useful tool to reflect soil N and P availability. Allocation to enzyme production can increase the availability of nutrient to uptake [23]. In the case of P, its availability in bulk soil depends on acid phosphatase activity (APA), in which plants and microbes produce acid phosphatase to hydrolyze organic P sources. APA has been shown to be tightly coupled with soil P availability, with an increase in APA typically indicating increased P limitation [24], [25].

To investigate the effects of N deposition and the potential role of P in mitigating N effects on forest ecosystem processes and functioning, a field experiment employing additions of N and P in a factorial design was initiated in 2007 in three lowland tropical forests in the Dinghushan Biosphere Reserve (DHSBR) in Southern China [26], [27], [28]. To our knowledge, there have been but four fertilization experiments in lowland tropical forests [29], [30], [31]. Our sites have lower available soil P (1.7 vs. 5 mg kg^–1^) and more acidic soil (pH 3.7 vs. 5.2) compared to that of a site in Panama [30], but similar to a Costa Rican forest [31]. The three forests in our study (one old-growth, and two younger) have contrasting soil N status, with the old-growth forest having larger N capital than the two younger forests [32], [33], allowing us to determine whether and to what extent soil N status influences ecosystem response to further N deposition and the role of P to mitigate effects of added N.

In the present study, we report responses of fine root biomass, the proportion of live fine roots, fine root nutrient concentrations, and APA in bulk soil to N and/or P treatments under the background of chronically-elevated atmospheric N deposition. Previous studies in the DHSBR from a long-term N addition experiment demonstrated a net loss of 8–16 kg N ha^–1^ yr^–1^ from the soil in the old-growth forest. In total, up to 60 kg N ha^–1^ yr^–1^ was leached from the old-growth forest, indicating that this forest was N saturated from chronic N deposition and long-term N accumulation [34]. Further N addition has been shown to increase soil acidification, base cation losses, and Al mobilization in this forest [9], [10]. In contrast, the two younger forests exhibit signs of N limitation, example.g., increased litter decomposition [32], litterfall production [35] and microbial activity [36] following N additions, although N leaching has also been observed [33]. We hypothesize that the old-growth forests have been facing strong P limitation due to long-term N accumulation, whereas the two younger forests are still N limited due to large plant N demand. If so, based on the allocation theory [17] and empirical studies, we expect that (1) N addition would decrease fine root biomass in all the three forests, through N-mediated soil chemical changes in the old-growth forest and through release from N limitation in the two younger forests, (2) P addition would increase fine root biomass in the old-growth forest while having no effects in the two younger forests.

## Materials and Methods

### Ethics Statement

No specific permits were required for the described field studies. This research station (Dinghushan Biosphere Reserve) belongs to South China Botanical Garden, Chinese Academy of Sciences. This study was also supported by this institute. Data will be made available upon request. We confirm that the location is not privately-owned or protected in any way. We also confirm that the field studies did not involve endangered or protected species.

### Study sites

The study was conducted in Dinghushan Biosphere Reserve (DHSBR) (112°33′E and 23°10′N) in the central section of Guangdong Province, southern China. The climate is warm and humid, with annual precipitation of 1927 mm and mean annual temperature of 21.0°C [37]. Soils are oxisols formed from sandstone [38]. In this area, high atmospheric N deposition has been on-going since 1990′s. Nitrogen deposition was 36 kg N ha^–1^ year^–1^ in 1990 and reached to 38 kg N ha^–1^ year^–1^ in 1999 [39], [40]. In 2004 and 2005, N deposition in rainfall measured was 34 and 32 kg N ha^–1^ year^–1^, respectively, 60% of which was in the form of NH_4_^+^-N [34].

Three forest types within the reserve (∼2–4 km apart) were used in this study: an old-growth (>400-yr-old) forest, considered the regional climax type, a younger pine forest, and a similarly younger mixed pine/broadleaf forest (∼75-yr-old; Table 1). All forest types are of similar elevation range (50–250 m above mean sea level), slope aspect and degree [33]. The notable age of the old-growth forest arises from long-term protection by monks from any form of direct human disturbance. The two younger forests originated from clear-cut harvesting and subsequent planting of *Pinus massoniana* in the 1930s, during which time the sites became badly eroded and degraded [10], [32]. Sharply contrasting intensity and frequency of litter and vegetation harvesting during 1930–1998 resulted in contrasting tree species composition. *Pinus massoniana* (*P. massoniana*) dominates the pine forest, whereas several broadleaf species co-dominate with *P. massoniana* the mixed forest. The three forest types differ greatly in N status, with greater N accumulation in the mineral soil and higher N leaching rates in the old-growth forest (Table 1).

**Table 1:** General characteristics of the three study forests.

	Old-growth	Pine	Mixed	References
**Aboveground**				
Age (year)	>400	∼75	∼75	[57]
Stem density (stems ha^–1^, DBH>2cm)	1013	767	1933	
Basal area (m^2^ ha^–1^)	26	14.0	13.8	
Litter production	7.1	6.0	6.1	[31]
(Mg ha^–1^ year^–1^) in 2009				
**Mineral soil (0–10 cm)**				
Bulk density (g cm^–3^)	1.0	1.2	1.2	[34]
Organic matter (%)	7.3	5.2	3.7	
Total N concentration (%)	0.2	0.12	0.1	
C:N	21	28	24	
Total P concentration (%)	0.05	0.04	0.04	
Net N mineralization	6.7	8.2	3.9	
(mg kg^–1^ month^–1^ of N)				
Net nitrification (mg kg^–1^ month^–1^ of N)	6.1	7.8	1.1	
Inorganic N leaching in 2006	41.4	20.3	8.9	[35]
(kg ha^–1^ year^–1^ of N, 20 cm below the organic layer)				

### Experimental design

A full 2×2 factorial was established in each forest type in 2007, with two levels (with and without addition) of each of two nutrients (N and P). Specifically, four treatments (each with 5 replicates), including control, N-addition (150 kg N ha^–1^ yr^–1^), P-addition (150 kg P ha^–1^ yr^–1^) and N+P-addition (150 kg N ha^–1^ yr^–1^ plus 150 kg P ha^–1^ yr^–1^) were set up in each forest. There were a total of 20 plots of 5 m×5 m in each forest and each plot was surrounded by a 5-m-wide buffer strip. Field plots were laid out randomly and were randomly selected to receive specific treatments. Plot size and fertilizer levels were chosen to resemble Cleveland and Townsend (2006) [41], who studied a tropical forest of Costa Rica. NH_4_NO_3_ or/and NaH_2_PO_4_ solutions were sprayed once every other month to the forest floor with a backpack sprayer starting from February 2007 and continued through July 2012. We considered our plots big enough to study soil processes and fine root dynamics, considering that root ingrowth core method is considered suitable to determine nutrient limitation [12], [14], [20].

### Fine root and soil sampling

We sampled fine roots from all plots in the three forests in July 2012 (about five years after the initiation of N and/or P treatments). In each plot, three 5-cm diameter cores of mineral soil were extracted randomly to a 10 cm in depth, then combined to yield one composite sample. From these samples, live and dead fine roots were distinguished by root resilience, brittleness, and color [42]. Roots were sorted, dried at 65°C, and weighed. Live fine root biomass, dead fine root biomass, and live fine root (biomass) proportion were determined. In July 2011, 0–10 cm mineral soil was sampled for analysis of soil chemical properties and APA. The soils were sieved to pass through 2 mm screen prior to chemical analysis.

### Chemical analysis

Acid phosphatase activity (APA) in one gram of fresh, sieved soil was analyzed according to the modified procedure by Scheneider et al. (2001) [43]. Concentration of NH_4_^+^-N was analyzed by the indophenol blue method followed by colorimetry, and NO_3_^–^-N was analyzed after cadmium reduction to NO_2_^–^, followed by sulfanilamide-NAD reaction [44]. Soil available P was extracted by acid-ammonium fluoride solution (0.025 mol L^–1^ HCL +0.03 mol L^–1^ NH_4_F) [45]. Soil pH was measured with soil: water ratio of 1:2.5 [44]. Oven-dried live fine root samples from each plot were ball milled before analyzed for total N concentration by EA-IRMS (EA1112 coupled with Delta-XP, Thermo Fisher Scientific K.K., Yokohama, Japan), and microwave digested with nitric acid before analyzed for total P concentration (inductively-coupled plasma emission spectrophotometry, Optima 2000, Perkin Elmer, USA).

### Statistical analysis

We calculated response ratios (RR) of all fine root and soil variables to N, P treatments as the experimental mean divided by the control mean, representing an index of response magnitudes; L_RR_ was calculated as the Ln of RR. Two-way analysis of variance (ANOVA) was used to examine the effects of N and P treatments and their interactions. Differences among forest types were compared using one-way ANOVA. All analyses were conducted using PASW STATISTICS 16.0 for Windows. Level of significance was set at *P*<0.05 unless otherwise stated.

## Results

### Fine root biomass

**Figure 1:**
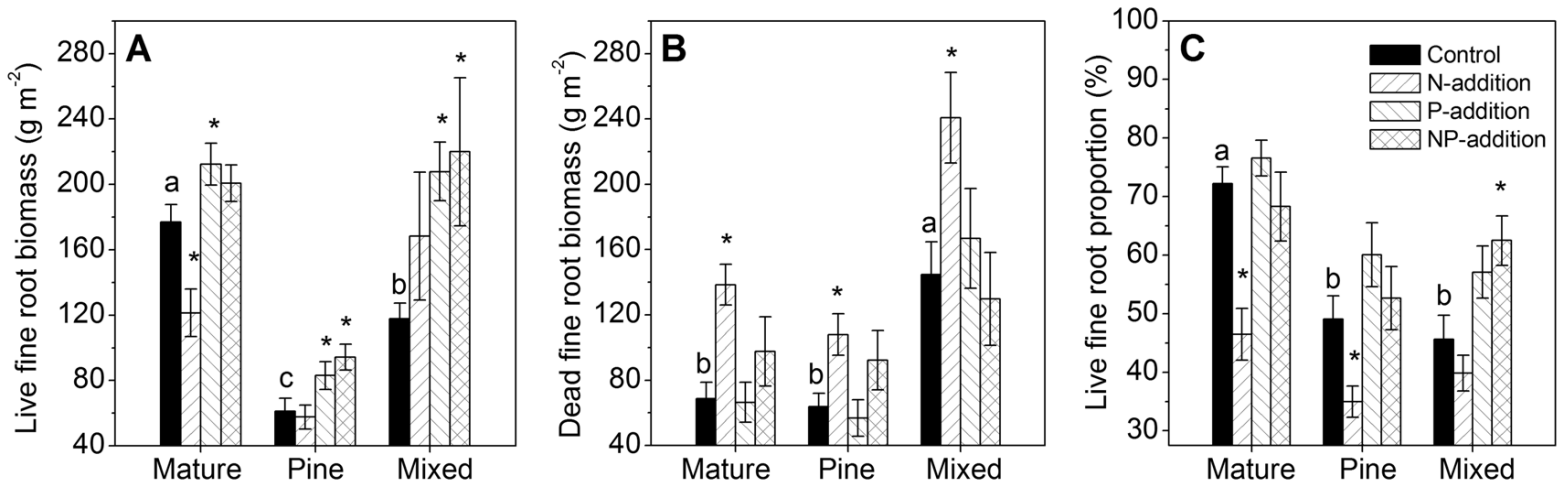
Live, dead fine root biomass and live fine root proportion after five years of nitrogen and phosphorus additions. Data from July 2012. Different letters indicate significant differences among forests (*P*<0.05). * indicates significant differences between each treatment and control (*P*<0.05). Error bars show SE (n = 5).

In the old-growth forest, N addition significantly reduced live fine root biomass (Fig. 1A; Table 2, 3) and significantly increased dead fine root biomass (Fig. 1B; Table 2, 3), resulting in a significant lower live fine root proportion, compared to respective values in control plots (Fig. 1C; Table 2, 3). In the two younger forests, compared to control plots, dead fine root biomass in N-addition plots was 69% higher in the pine forest (108.0 g m^–2^ vs. 63.8 g m^–2^, Fig. 1; Table 2, 3), and 66% higher in the mixed forest (240.6 g m^–2^ vs. 144.6 g m^–2^, Fig. 1). No significant effects of N addition on live fine root biomass or live fine root proportion were observed in the two younger forests, except that live fine root proportion in the pine forest was significantly lower in N-addition plots (Fig. 1A,C; Table 2, 3).

**Table 2:** Response ratios of fine root and soil variables to N and P treatments.

Forests/Treatments	Old-growth			Pine			Mixed		
	N-addition	P-addition	NP-addition	N-addition	P-addition	NP-addition	N-addition	P-addition	NP-addition
Live fine root biomass	−0.38*	0.18*	0.13	−0.06	0.30*	0.43*	0.36	0.57*	0.62*
Dead fine root biomass	0.70*	–0.04	0.35	0.53*	−0.12	0.37	0.51*	0.14	−0.11
Live proportion	−0.44*	0.06	−0.05	−0.34*	0.20	0.07	−0.14	0.22	0.31*
Fine root N	0.05	−0.24	−0.12	0.01	−0.2	0.02	0.02	0.08	0.01
Fine root P	0.29	1.09*	0.85*	0.22	0.92*	1.01*	0.41	1.39*	1.39*
N:P	−0.09	−1.17*	−0.73*	0.12	−0.98*	−0.78*	−0.52	−1.47*	−1.57*
pH	−0.02*	0.03*	0.01	0.01	−0.01	−0.02*	0.01	0.03*	0.02
Available P	0.11	2.85*	2.65*	0.56	3.47*	3.29*	−0.25	2.72*	2.60*
NH_4_^+^-N	−0.48	0.25	−0.40	−0.44	−0.20	−0.17	−0.01	−0.34	−0.56
NO_3_^−^-N	0.46*	−0.43	−0.43	−0.10	−0.35	−0.27	−0.31	−0.61	−0.41
APA	0.21*	−0.40*	−0.37*	0.01	−0.16	−0.26	−0.05	−0.30*	−0.95*

Values are Ln(treatment mean/control mean). A positive response ratio indicates positive effects of fertilization, whereas a negative one indicates negative effects. * indicates significant difference at *P*<0.05.

**Table 3:** Summary of results of two-way ANOVA. Significant effects indicated by *, ** and ***, representing probability at the 5%, 1% and 0.1% levels, respectively; ns, not significant.

	Two-way ANOVA								
Forests/Treatments	Old-growth			Pine			Mixed		
	N	P	N×P	N	P	N×P	N	P	N×P
Live fine root biomass	*	**	ns	ns	**	ns	ns	*	ns
Dead fine root biomass	**	ns	ns	**	Ns	ns	ns	ns	ns
Live fine root proportion	**	**	ns	**	**	ns	ns	**	ns
Fine root N	ns	ns	ns	ns	Ns	ns	ns	ns	ns
Fine root P	ns	**	**	ns	**	ns	ns	**	ns
N:P	ns	***	ns	ns	***	ns	**	***	ns
pH	***	***	ns	ns	Ns	ns	ns	**	*
Available P	ns	***	ns	ns	***	ns	ns	***	ns
NH_4_^+^-N	ns	ns	ns	ns	Ns	ns	ns	*	ns
NO_3_^−^-N	ns	**	ns	ns	Ns	ns	ns	ns	ns
APA	*	***	ns	ns	Ns	ns	ns	***	**

Plots with added P had elevated live fine root biomass in all three forests. Specifically, P addition resulted in increases of 20% in the old-growth forest (212.4 vs. 176.9 g m^–2^, Fig. 1A; Table 2, 3), of 35% in the pine forest (83 vs. 61.2 g m^–2^, Fig. 1A; Table 2, 3) and of 76% in the mixed forest (207.8 vs. 117.8 g m^–2^, Fig. 1A; Table 2, 3), compared to respective controls. Two-way ANOVA showed that P treatments had no significant effects on dead fine root biomass in any forest, but significantly altered live fine root proportion in all three forests (Table 2, 3; Fig. 1B, C).

N and P treatments had no interactive effects on neither live nor dead fine root biomass, or on live fine root proportion in any forest (Table 3). N+P treatment in the two younger forests significantly increased live fine root biomass relative to control (by 74% in the pine forest and by 87% in the mixed forest, Fig. 1A; Table 2), similar to the effects of P addition alone. Higher live fine root proportion in N+P-addition plots comparing to control was also observed in the mixed forest (Fig. 1C; Table 2).

### Fine root nutrient concentrations

**Figure 2:**
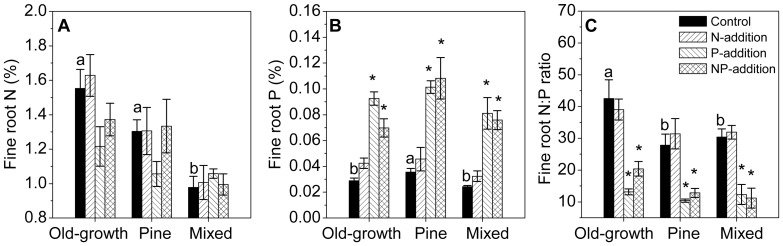
Nutrient concentration in live fine roots after five years of nitrogen and phosphorus additions. Data from July 2012. Different letters indicate significant differences among forests (*P*<0.05). * indicates significant differences between each treatment and control (*P*<0.05). Error bars show SE (n = 5).

The old-growth forest had significantly higher fine root N concentrations and N:P ratios than the two younger forests (Fig. 2A, C). Fine root P concentration was highest in the pine forest (Fig. 2B). N treatments did not alter N or P concentrations in any forest (Fig. 2; Table 2, 3). P treatments had no significant effects on fine root N concentrations (Fig. 2A; Table 3), but significantly elevated fine root P concentrations in all three forests, by 2 to 4 folds (Fig. 2B; Table 2, 3). N:P ratios of fine roots were significantly lower in P-addition and NP-addition plots in all three forests, compared with those in control (Fig. 2C). There were interactions between N and P treatments on fine root P in the old-growth forest (Fig. 2B; Table 3).

### Soil chemical properties

**Figure 3:**
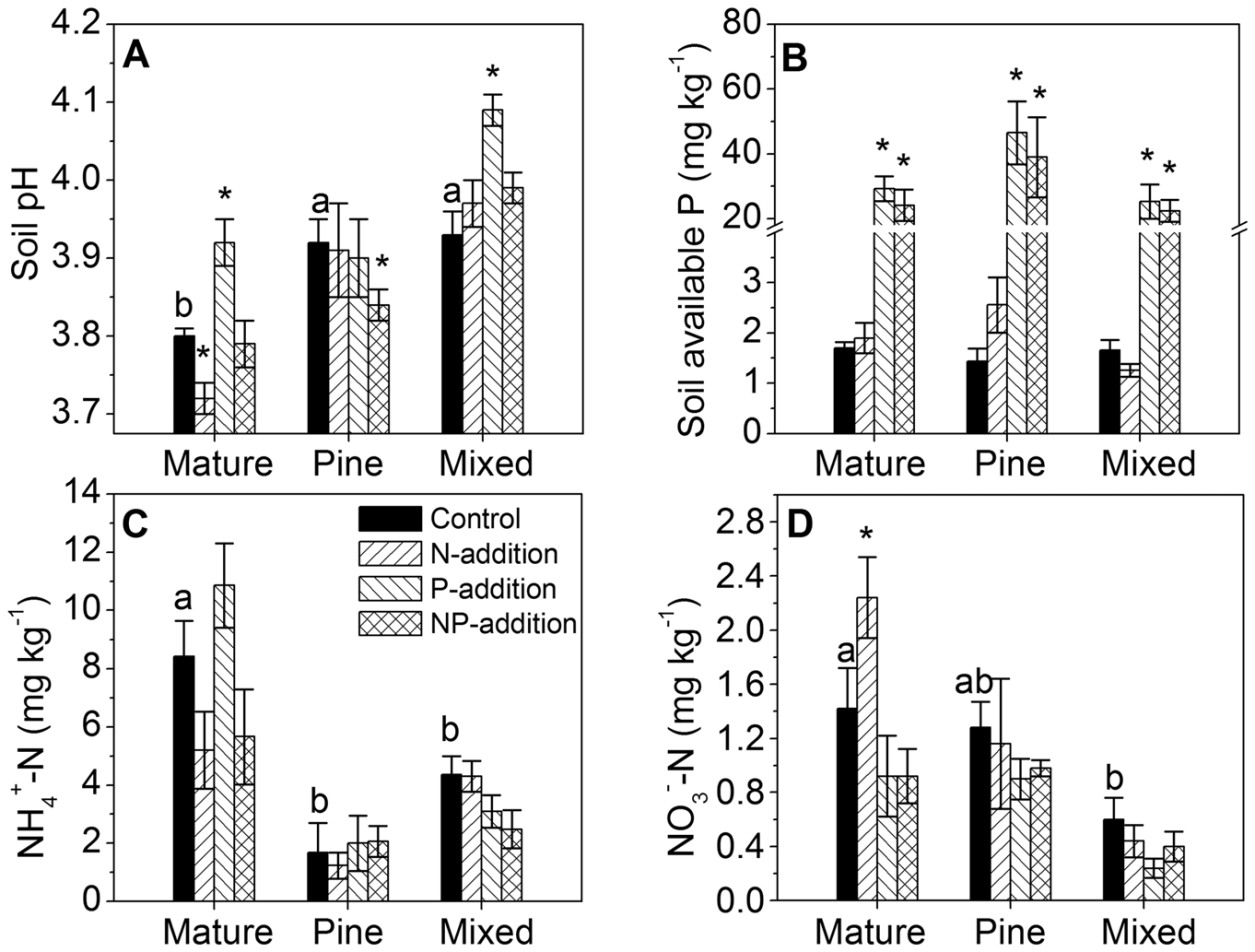
Soil pH, available P and N (NH_4_^+^-N and NO_3_^−^-N) after four years of nitrogen and phosphorus additions. Data from July 2011. Different letters indicate significant differences among forests (*P*<0.05). * indicates significant differences between each treatment and control (*P*<0.05). Error bars show SE (n = 5).

Soils in all the three forests were acidic (pH <4.0), with the lowest pH in the old-growth forest (Fig. 3A). The old-growth forest was significantly higher in soil NH_4_^+^-N and NO_3_^–^-N than the other two forests, whereas soil available P concentrations did not vary among the three forests (Fig. 3B–D).

Soil pH was lower in N-addition plots than in control in the old-growth forest, but not in the two younger forests (Fig. 3A; Table 2, 3). N treatments had no significant effects on soil available P or available N (NH_4_^+^-N and NO_3_^–^-N) in any forest, except that in the old-growth forest, NO_3_^–^-N in N-addition plot was significantly higher than in control (Table 2, 3; Fig. 3B–D). In contrast, P treatments significantly increased soil pH in the old-growth forest and the mixed forest (Table 2, 3; Fig. 3A), but not in the pine forest. Soil available P increased significantly following P addition by 16-, 31-, and 14-fold, and following NP additions by 13-, 26-, and 12-fold in the old-growth, pine, and mixed forests, respectively, compared with respective controls (Fig. 3B). P treatments decreased NO_3_^−^ in the old-growth forest and decreased NH_4_^+^-N in the mixed forest (Table 3). Interaction between N and P only existed for soil pH in the mixed forest (Table 3).

### APA in bulk soil

**Figure 4:**
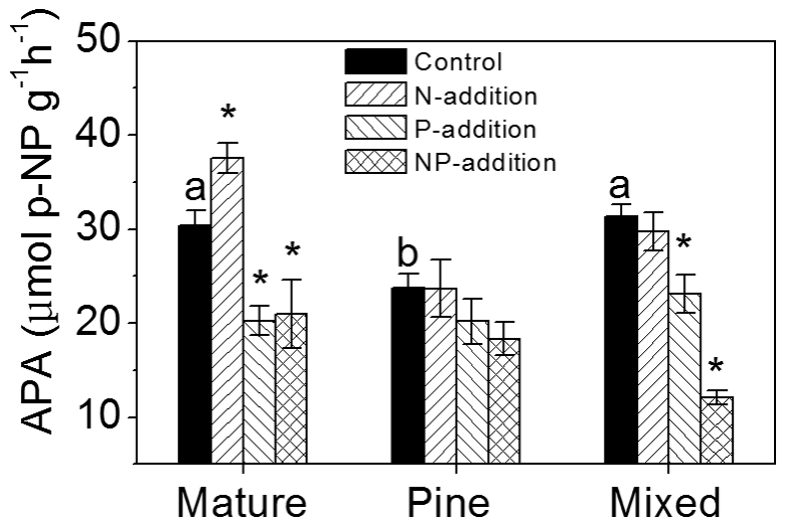
Acid phosphatase activity (APA) in bulk soil after four years of nitrogen and phosphorus additions. Data from July 2011. Different letters indicate significant differences among forests (*P*<0.05). * indicates significant differences between each treatments and control (*P*<0.05). Error bars show SE (n = 5).

APA in control plots was significantly lower in the pine forest than in the other two forests (Fig. 4). Effects of nutrient treatments on APA varied with forest type. Significantly higher APA in N-addition plots compared with that in control was only observed in the old-growth forest (Fig. 4; Table 2). In contrast, P and N+P treatments resulted in declines in APA in the old-growth and the mixed forests, but not in the pine forest (Fig. 4; Table 2). N+P treatments had interactive effects on APA only in the mixed forest, exhibiting synergistically inhibitive effects (Table 3).

## Discussion

### Contrasting responses of fine roots to N additions in three tropical forests

The main purpose of this study was to examine the nutrient status of three lowland tropical forests in southern China by quantifying the response of fine roots and APA to five years of N and P additions. Based on the different ages of these forests and their responses to N addition in another N manipulation experiment in DHSBR [8], [32], we hypothesized that the old-growth forest was N saturated, whereas productivity in the two younger forests was still N limited, despite chronically elevated N deposition. Thus, we expected N addition to decrease fine root biomass and live fine root proportion (vitality) in the old-growth forest through deleterious changes in soil properties (e.g., increased soil acidification and Al mobilization) [9], [10], and in the two younger forests through less carbon allocation to fine roots driven by additional N [18], [19].

The results from the old-growth forest support our expectation, in that five years of N addition at 150 kg N ha^–1^ yr^–1^ significantly decreased live fine root biomass and increased dead fine root biomass, resulting in decreased live fine root proportion (vitality). Whereas a decrease in total fine root biomass following two years of N additions had been observed in another N manipulation experiment in DHSBR [8], our results added further evidence of the adverse effects of N on fine root growth in this forest, i.e., different directions of changes in live and dead fine root biomass, and a decreased live fine root proportion, indicative of damage to fine roots [46].

Elevated N input can lead to soil acidification [7], [10], which has been associated with significantly lower fine root vitality (live fine root proportion) than that from less acidified soils [47]. In Norway spruce stands in Sweden, acidification caused by addition of ammonium sulfate resulted in faster rates of root death [48], [49]. Our results from the old-growth forest supported this observation, with soil pH decreasing significantly following N additions (Fig. 3A). A decline of live fine root biomass and a concurrent increase of dead fine root biomass after N additions have also been observed in other wet tropical forests [50], [51], with authors attributing these results to either direct effects of N on belowground root growth/turnover, or to a secondary effect of N fertilization on belowground plant dynamics (e.g. decline in soil pH [50]).

Contrary to our expectations, we failed to observe decreases in fine root biomass following N additions in the two younger forests. Instead, we found increased dead fine root biomass and reduced live fine root proportion in N-addition plots in the two younger forests, which were similar to the observation in the old-growth forest. It appeared that five years of N additions may have moved these forests toward N saturation, when negative secondary effects of N on fine root growth started to emerge. Previous disturbance before the 1990′s through understory vegetation and litter harvesting lowered the ecosystem N capital relative to the undisturbed old-growth forest [32]. Furthermore, N additions failed to alter fine root N or P concentrations in any of these three forests, consistent with other studies in tropical forests [16].

The pattern of APA response to N in the three forests paralleled the response of live fine root biomass, with significant increase of APA observed only in the old-growth forest (Fig. 4). Increase of APA suggests further demand for P driven by N addition to the N saturated forest [24], [52]. Elevated APA stimulated organic P mineralization and released inorganic P, which in turn likely compensated the low soil P availability. This may explain why available soil P in N-addition plots did not vary significantly from control in the old-growth forest, albeit both were of very low levels (Fig. 3B). In the two younger forests, N addition had no effects on APA or soil pH, suggesting addition of 150 kg N ha^–1^ yr^–1^ did not drive P demand to the extent to induce APA. Thus, they may not be limited by P as the N-saturated old-growth forest. Lower N status may also have constrained phosphatase production, which is highly N-consumptive [25]. Collectively, these results suggest that initial soil N status of the study forests largely influenced fine root responses to increased N inputs.

### Variation in P limitation for fine root growth among forests

Following the criteria to assess nutrient limitations stated by Vitousek et al. (2010) [15], we further address P limitation for fine root growth and alleviation of P limitation by P fertilization at the three study forests by combining direct and indirect measurements of (1) fine root growth in response to P fertilization, (2) fine root nutrient concentrations, and (3) nutrient availability and APA in bulk soil.

Contrary to expectation, fine root growth responded positively to P treatments in all three study forests, albeit to a larger extent in old-growth forest and mixed forests, suggesting P limitation to fine root growth in these forests. This pattern corresponded well with notably elevated soil P availability in these forests, indicating that fine roots likely accessed elevated P by increasing growth into the surface soil (0–10 cm) [12]. Significantly higher fine root biomass and fine root N uptake following P additions had been observed in an N-saturated Mediterranean-fir (*Abies pinsapo*) forest in southern Spain which exhibited symptoms of P limitation [53]. A trend toward higher fine root biomass after two years of N+P additions was also observed in the ongoing fertilization experiment in lowland tropical forests in Costa Rica [31]. Fine root biomass represents a balance between fine root production and decomposition. Fine root decomposition, which is microbially mediated [16], may have been stimulated in our study forests, because microbial activities were found to be significantly higher in P-addition plots in the old-growth and the mixed forests [27]. Thus, the observed increases of live fine root biomass and no changes in dead fine root biomass indicate the likelihood of enhanced fine root production following P additions in these forests, which has also been shown in other tropical forests. In an old site (>4 million years) in Hawaii where aboveground net primary production was P-limited, both root net primary production and root turnover (decomposition) increased following P fertilization [16]. In a montane tropical forest, moderate N and P additions enhanced fine root production and turnover at the same time, resulting in a reduced fine root biomass [51]. A meta-analysis research which synthesized data from N and P gradients showed that in lowland tropical forests, fine root production was positively associated with both natural and artificial P gradients [54].

P addition improved fine root nutrient condition. Plant nutrient imbalance can be expected in these forests, with higher fine root N (average 13 vs. 11 g kg^–1^), but much lower P (average 0.3 vs. 0.9 g kg^–1^) compared with the global average values compiled by Gordon and Jackson (2000) [55]. Fine root N:P ratios are as high as 43, 28, and 30 in the old-growth, pine and mixed forests, respectively, suggesting high demand of P for tree growth. These ratio values are much higher than the averaged value of 12 from the compiled data set [55], than the values of 26, 17, and 23 of live fine roots in the 300 yr-, 20000 yr- and >4 million yr-old forests along the geologic chronosequence in Hawaii [16], and also higher than the threshold of foliar N:P ratio of 20 beyond which P limitation is suggested [56]. Notably, substantial improvement of fine root nutrient conditions by P addition was observed, i.e., fine root N:P ratios were altered by P addition from high levels of 43, 28, and 30 to 13, 10, and 12 in the old-growth, pine and mixed forests, respectively, which were largely contributed by elevated fine root P concentrations (Fig. 2B, C).

Notably, in spite of high fine root P uptake after P fertilization in all three forests, fine root N concentrations showed no response to P additions. This contrasts with results of Blanes et al. (2012) [53], who found that P addition to an N-saturated Mediterranean-fir (*Abies pinsapo*) forest increased the competitive ability of tree roots for soil N. In our experiment, however, higher microbial N was found following P addition in the old-growth forest [28], implying potentially higher microbial immobilization of P and N. Available soil P in our sites (∼2 mg kg^–1^) was much lower than in the soil of Mediterranean-fir (*Abies pinsapo,* 7.8 mg kg^–1^), suggesting that the competition between tree roots and free-living microbes for available P may be greatly enhanced in our study sites.

## Conclusions

Five years of N and P treatments in relatively high amounts (150 kg ha^–1^ yr^–1^) substantially altered fine root biomass, nutrient concentrations, APA of bulk soil, and other soil chemical properties in the three study forests. As hypothesized, these forests exhibited diverse responses to N and/or P treatments, largely reflecting contrasting N status. In contrast, fine root growth appeared to be P-limited in all three forests, with the N saturated old-growth forest being more P-limited than the other two forests, based on its APA responses to N addition. These patterns suggest that initial soil nutrient status have large impact on potential responses of belowground structure to further nutrient inputs in these ecosystems. While high N deposition in this region keeps adding N to these tropical forests, P limitation emerges and becomes a key factor to influence ecosystems processes.
